# Geboes Histopathology Score Grade 3 Subclassification Predicts Treatment Response in Active Ulcerative Colitis

**DOI:** 10.3390/jcm14207214

**Published:** 2025-10-13

**Authors:** Ichitaro Horiuchi, Akira Horiuchi, Kaori Horiuchi, Shun Watanabe, Tsuyoshi Terashima, Ken Sugimoto

**Affiliations:** 1 Department of Gastroenterology, Shinshu University School of Medicine, Matsumoto 390-8621, Japan; ichitaro617@yahoo.co.jp; 2 Digestive Disease Center, Showa Inan General Hospital, Komagane 399-4117, Japan; koraki648@yahoo.co.jp (K.H.); shunnnnnn7@gmail.com (S.W.); 3 Department of Pathology, Showa Inan General Hospital, Komagane 399-4117, Japan; tera.tsuyo@gmail.com; 4 First Department of Internal Medicine, Hamamatsu Medical University, Hamamatsu 431-3192, Japan; sugimken@hama-med.ac.jp

**Keywords:** ulcerative colitis, IL23, Geboes Score Grade 3, IL-23p19 inhibitor, personalized medicine

## Abstract

**Background:** The growing number of ulcerative colitis (UC) treatments has complicated the selection process. We hypothesized that the degree of epithelial neutrophilic infiltration, which is a hallmark of interleukin-23 (IL-23) pathway activation, could guide personalized therapy. **Methods:** This single-center, prospective study included 42 patients with active UC who were treated between July 2024 and September 2025. The study was registered (NCT 06626165) on 20 July 2024. Prior to treatment, all patients underwent colonoscopy with biopsy. Using a refined subclassification of the Geboes histopathology score, patients were stratified into two groups based on the percentage of crypts with neutrophilic infiltration: Grade ≥ 3.2 and Grade < 3.2. The primary outcome was clinical remission at four weeks. The secondary outcome was endoscopic remission at six months. **Results:** Of the 42 patients, 22 were classified as Grade ≥ 3.2, and 20 were classified as Grade < 3.2. Baseline clinical and endoscopic characteristics were similar between the two groups. All 22 patients in the Grade ≥ 3.2 group treated with mirikizumab achieved clinical remission within four weeks. Nineteen (86%) of the twenty-two patients achieved endoscopic remission within six months. All 20 patients in the Grade < 3.2 group who received vedolizumab (16) or upadacitinib (4) achieved clinical remission within the same timeframe. Both treatment strategies led to a statistically significant reduction in the inflammatory biomarker leucine-rich alpha-2-glycoprotein (LRG) (*p* < 0.001). **Conclusions:** Our findings suggest that UC patients with advanced epithelial neutrophil infiltration (Geboes Grade ≥ 3.2) may have a disease that is predominantly driven by the IL-23 pathway, making them potential candidates for selective IL-23p19 inhibitors. This histopathology-driven approach appears to be a useful strategy for tailoring treatment to patients with active UC, though further validation is required.

## 1. Introduction

Ulcerative colitis (UC) is an inflammatory disease of the colon, and its pathogenesis remains unclear. The disease is characterized by a prolonged course of relapse and remission, significantly impairing patients’ quality of life [1,2]. Over the past two decades, treatment options for moderate to severe UC have expanded remarkably [1,3]. Various molecular targeted therapies are now available for patients who are steroid-dependent or steroid-refractory [2]. Furthermore, UC management has been revolutionized by the treat-to-target approach, which aims to achieve predefined therapeutic goals to improve long-term outcomes [4,5]. The range of therapeutic choices has rapidly broadened to include agents with different mechanisms of action, such as TNF-α inhibitors, integrin antagonists, Janus kinase inhibitors, interleukin (IL)-12/23 and IL-23p19 inhibitors, and sphingosine-1-phosphate modulators [2,6]. While this expansion offers more options, primary non-response (lack of efficacy of initial therapy) remains a common issue, making the decision-making process for selecting optimal initial therapy increasingly important [2,6].

Among the major immunological pathways in UC, the IL-23/Th17 axis is recognized as a central driver of inflammation. IL-23 activates Th17 cells, which promote the production of pro-inflammatory cytokines and mobilize and activate neutrophils [7,8]. Neutrophil infiltration into the colonic mucosal epithelium plays a critical role in tissue damage and inflammation in UC and serves as an important histopathological marker for assessing disease activity [9]. The Geboes histopathological score is widely used to evaluate inflammation in UC, with grade 3 specifically assessing neutrophil infiltration into the epithelium [10]. However, the original description lacked a precise methodology for quantifying this infiltration, which could result in inter-observer variability. In our previous study, we developed a concise and clearly defined scoring method for Geboes Score Grade 3 subclassification, based on its original definition, which demonstrated high inter-pathologist consistency and reproducibility [11].

This study aims to evaluate a treatment strategy for selecting optimal initial therapy based on this refined Geboes Score Grade 3 scoring method. We hypothesized that patients with high epithelial neutrophil infiltration (Grade ≥ 3.2) have disease primarily driven by the IL-23 pathway and are likely to respond well to selective IL-23p19 inhibitors. Conversely, patients with lower levels of infiltration may benefit from agents with different mechanisms of action. We report the treatment outcomes of a UC patient cohort managed according to this histology-based algorithm.

## 2. Materials and Methods

### 2.1. Study Design and Patients

**Figure 1:**
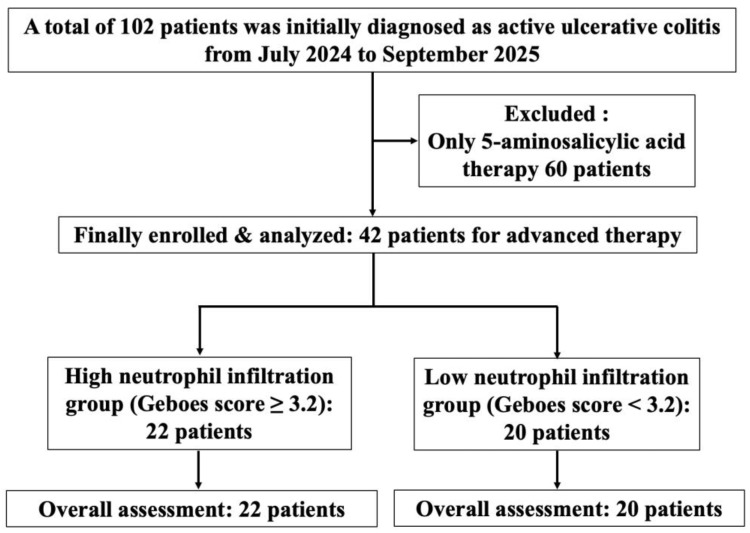
Study flow diagram of enrolled patients.

This single-center, prospective, observational cohort study was conducted at Showa Inan General Hospital in Komagane, Japan, from July 2024 to September 2025. A total of 102 patients were initially diagnosed with active UC. Of those patients, 60 who received only 5-aminosalicylic acid therapy were excluded. Finally, 42 patients who received treatment based on histological assessment were included (Figure 1). The study protocol (No. 2023-7) was approved by our hospital’s institutional ethics committee, and all procedures adhered to the Declaration of Helsinki. All patients provided written informed consent prior to enrollment in the study. The study was registered at www.clinicaltrials.gov (NCT 06626165) on 1 October 2024.

### 2.2. Clinical, Endoscopic, and Histopathological Evaluations

For the evaluation of UC, we used a clinical activity index (CAI) to determine each patient’s clinical activity [12]. Bowel urgency was evaluated using a self-reported numeric rating scale (NRS) [13]. Each patient’s extension of UC disease was classified as proctitis, left-sided colitis, or pancolitis. The patients’ endoscopic activity was assessed using the Mayo endoscopic subscore (MES), with an MES score ≥ 1 point indicating active UC [14]. The Ulcerative Colitis Endoscopic Index of Severity (UCEIS) score was calculated by summing the scores of three factors, assessing the site with the maximum endoscopic activity: the vascular pattern (0–2 points), bleeding (0–3 points), and erosions and ulcers (0–3 points) [15]. Pathological activity was also graded using the Geboes biopsy histology scores on a scale from 0 to 5, with higher scores indicating more severe histologic inflammation [10]. Prior to initiating induction therapy, all patients underwent a colonoscopy where biopsies were taken from the most inflamed area of the mucosa. The biopsy specimens were stained with hematoxylin and eosin (H&E). Additionally, a colonoscopy was performed on all patients six months after induction therapy.

### 2.3. Komagane Evaluation Method

**Figure 2:**
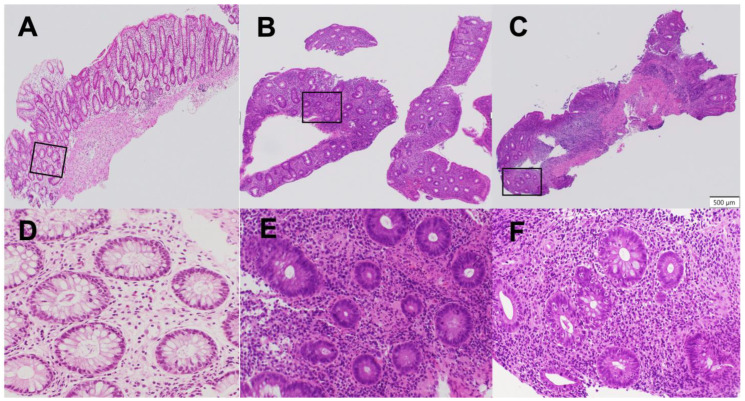
Assessment of Geboes score Grade 3. The percentage of crypts with neutrophilic infiltration was calculated as the number of crypts with neutrophilic infiltration/the total number of crypts on a glass slide, and the black frame indicates the high resolution area. This was used to subclassify the Geboes score Grade 3 into Grades 3.1 3/132 = 2.3% ((**A**) low resolution; (**D**) high resolution), Grades 3.2 22/125 = 17.6% ((**B**) low resolution; (**E**) high resolution), and Grades 3.3 25/36 = 69.4% ((**C**) low resolution; (**F**) high resolution).

Epithelial neutrophilic infiltration was quantified using a refined subclassification of the Geboes Grade 3 score, termed the “Komagane evaluation method” [11]. The methodology was as follows: First, the total number of crypts on a glass slide was counted. Second, a crypt with neutrophilic infiltration was defined as containing at least two neutrophils within the crypt epithelium or lumen. Third, the percentage of involved crypts was calculated, and a score was assigned: Grade 3.1 (less than 5% of crypts involved), Grade 3.2 (less than 50% involved), or Grade 3.3 (more than 50% involved) [10]. Patients were divided into two treatment groups based on this score: the high infiltration Group (Geboes Score ≥ 3.2) and the low infiltration Group (Geboes Score < 3.2) (Figure 2).

### 2.4. Raters and Reliability Testing

Two raters performed the Komagane evaluation method of the Geboes score Grade 3. The raters had different durations of experience in performing and interpreting histological evaluations (28 years for the expert rater and 1 year for the beginner rater). Both raters had extensive training (including written definitions, visual depictions, and verbal explanations) regarding the reliable and consistent use of the Komagane evaluation method for Geboes score Grade 3. The intra-rater test–retest reliability, the inter-rater reliability, and the construct validity were evaluated for the Komagane evaluation method for Geboes score Grade 3, which was obtained for all glass slides by the same two raters, 4 weeks apart, and with the order of slide presentations randomized. The intra-rater test–retest reliability for the Komagane evaluation method, assessed four weeks apart, was determined using a weighted Kappa statistic. The analysis showed perfect agreement for both the expert rater (κ = 1.0) and the beginner rater (κ = 1.0), indicating excellent consistency of the scoring method for each individual evaluator. The construct validity was also 100% for both raters. The inter-rater reliability between the two raters for the Komagane evaluation method was substantial, with a multi-rater Kappa value of 0.82.

### 2.5. Treatment Stratification Based on the Komagane Subclassification of the Geboes Score Grade 3

In the high neutrophil infiltration group (Geboes score ≥ 3.2), mirikizumab was chosen because of its selective inhibition of IL-23p19, which we hypothesized would be particularly effective. Risankizumab, another IL-23p19 inhibitor that acts similarly, was unavailable in Japan when the study began in July 2024. The first six patients enrolled in this study were treated with 300 mg of mirikizumab intravenously every 4 weeks for 12 weeks [16]. The remaining 16 (73%) of the 22 patients received 300 mg of mirikizumab administered intravenously every 4 weeks for 24 weeks. As maintenance therapy, all 22 patients received 200 mg of mirikizumab administered subcutaneously every 4 weeks.

The low neutrophil infiltration Group (Geboes Score < 3.2) received either the α4β7 integrin monoclonal antibody, vedolizumab, or the Janus kinase inhibitor, upadacitinib. Upadacitinib was selected for patients with UC at CAI ≥ 12, and vedolizumab was used for patients below that level. Vedolizumab is administered as a 300 mg intravenous infusion at weeks 0, 2, and 6, followed by maintenance infusions of 300 mg every eight weeks thereafter. Upadacitinib is taken orally at 45 mg once daily for eight weeks during induction, then 15 mg once daily for maintenance therapy. Seventeen of the forty-two enrolled patients were taking oral steroids (10 mg/day or less) at the beginning of the study; however, they discontinued the steroids before receiving the first dose of the investigational treatment. These patients were considered steroid-dependent because they had been unable to taper off steroids successfully in previous attempts. The remaining 25 patients were steroid-free at the time of enrollment. Patients receiving a stable maintenance dose of 5-aminosalicylic acid therapies prior to enrollment were allowed to continue their treatment without dose adjustments.

### 2.6. Measurements of Serum C-Reactive Protein (CRP) and Leucine-Rich α2 Glycoprotein (LRG) Levels

Blood tests and a colonoscopy with histology were performed on the same day. The patients’ CRP levels were measured using the latex immunoturbidimetric method. Their serum LRG levels were measured by latex turbidimetric immunoassay using a commercially available kit (Sekisui Medical, Tokyo, Japan) [17,18,19].

### 2.7. Safety Monitoring

Patient safety was monitored throughout the six-month study. Patients were instructed to report any signs of infection, reactions at the infusion or injection site, or other new symptoms. Assessments included physical examinations and laboratory tests, such as a complete blood count, liver function tests, CRP, and LRG. Adverse events were recorded at each visit.

### 2.8. Outcome Measures

The primary outcome was clinical remission (CAI < 4) [12], which was evaluated four weeks after induction therapy with each agent. The secondary outcome was endoscopic remission (MES 0, UCEIS 0) [14,15] six months after induction therapy. Other outcome measures included changes in hemoglobin, albumin, CRP, and LRG before and four weeks after induction therapy.

### 2.9. Statistical Analysis

Data are presented as mean ± SD or as median (25th, 75th percentile). Categorical data were compared using the χ^2^-test, with Yates’ correction applied when appropriate, or Fisher’s exact test for smaller sample sizes. Student’s t-test was used to compare the means of parametric data, while the Mann–Whitney rank sum test was used for comparing medians of non-parametric data. A *p*-value < 0.05 was deemed statistically significant. All statistical analyses were carried out using GraphPad Prism software (v. 10.2.3) (GraphPad, Boston, MA, USA).

## 3. Results

### 3.1. Patients’ Clinical Characteristics

A total of 42 patients were included, with a mean age of 44 years; 23 (55%) were female. Twenty-two patients were classified into Geboes ≥ 3.2 group, and 20 were Geboes < 3.2 group. Baseline characteristics, including disease duration and prior treatment history, were well-matched between the groups (Table 1). The disease extent was pancolitis in 22 cases and left-sided colitis in 20 cases. There were no statistically significant differences in age, sex, disease duration, disease extension, or prior exposure to biologics or small molecules. Baseline disease activity, measured by CAI, NRS, MES, and UCEIS, was also similar between the groups. Prior treatments included conventional therapies, as well as advanced medications, such as infliximab, ustekinumab, and filgotinib. No patients in the cohort had significant comorbidities that would have affected the selection of therapy or the interpretation of the results. At study entry, four patients (9.5%) were hospitalized, and one patient (2.4%) had visited the emergency department.

### 3.2. Changes in Blood Biomarkers

Significant improvements were observed in inflammatory markers. In the Geboes ≥ 3.2 group, mean LRG levels decreased from 18.3 ± 3.6 µg/mL to 11.0 ± 1.9 µg/mL (*p* < 0.001) after 4 weeks of treatment with mirikizumab. In the Geboes < 3.2 group, mean LRG levels decreased from 20.3 ± 4.1 µg/mL to 15.0 ± 3.1 µg/mL (*p* < 0.001) after 4 weeks of treatment with vedolizumab or upadacitinib. Changes in hemoglobin, albumin, and CRP trended towards improvement but did not reach statistical significance in this small cohort (Table 2).

### 3.3. Treatment Efficacy

**Figure 3:**
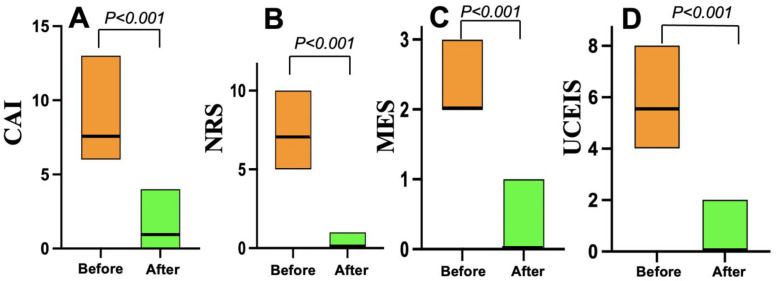
Changes in clinical and endoscopic scores before and after mirikizumab treatment in the Geboes Grade ≥ 3.2 group (*n* = 22). (**A**) The clinical activity index (CAI) scores. (**B**) The numeric rating scale (NRS) for bowel urgency values. (**C**) The Mayo endoscopic subscore (MES) data. (**D**) The Ulcerative Colitis Endoscopic Index of Severity (UCEIS) scores.

**Figure 4:**
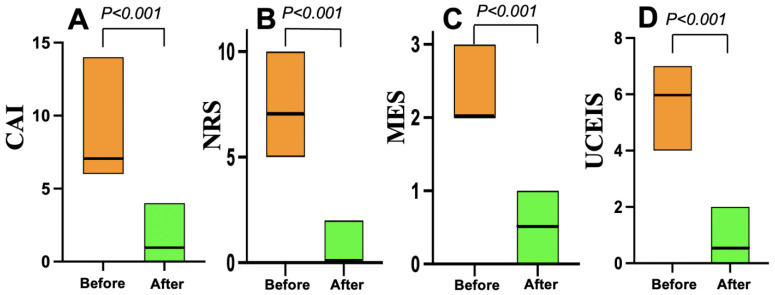
Changes in clinical and endoscopic scores before and after vedolizumab or upadacitinib treatment in the Geboes Grade < 3.2 group (*n* = 20). (**A**) The clinical activity index (CAI) scores. (**B**) The numeric rating scale (NRS) for bowel urgency values. (**C**) The Mayo endoscopic subscore (MES) data. (**D**) The Ulcerative Colitis Endoscopic Index of Severity (UCEIS) scores.

As shown in Figure 3, all 22 patients in the Grade ≥ 3.2 group treated with mirikizumab achieved clinical remission within four weeks. Nineteen (86%) who received 300 mg of mirikizumab administered intravenously every 4 weeks for 12 or 24 weeks achieved endoscopic remission within six months. On the other hand, all 20 patients in the Grade < 3.2 group who received vedolizumab (16) or upadacitinib (4) achieved clinical remission within the same timeframe, but 10 (50%) of the 20 patients achieved endoscopic remission within six months (Figure 4).

### 3.4. Safety and Adverse Events

During the six-month observation period of this study, no adverse events, including infections or infusion-related reactions, were recorded in either treatment group, and no patients discontinued treatment due to adverse events.

## 4. Discussion

This study demonstrates the potential utility of a histology-guided treatment algorithm for active UC. By stratifying patients based on the intensity of epithelial neutrophilic infiltration, we achieved a 100% clinical remission rate at 4 weeks and 86% endoscopic remission at 6 months in all 22 patients in the Grade ≥ 3.2 group treated with mirikizumab. These results suggest that subclassification of Geboes Grade 3 may serve as a practical biomarker for individualizing therapy.

An important finding was the high rate of endoscopic remission (86%) observed in the Geboes Grade ≥ 3.2 group. These results differ markedly from the pivotal LUCENT-1 trial [16], which reported lower remission rates. While the longer 24-week induction regimen used for most of our patients may have contributed to this outcome, we believe the primary driver of this enhanced efficacy is our histology-based patient selection. By identifying a patient population whose disease is characterized by intense neutrophilic infiltration—a likely marker of IL-23 pathway activation—we were able to enrich for patients most likely to respond to targeted IL-23p19 inhibition.

In addition, the significant reduction from 18.3 ± 3.6 to 11.0 ± 1.9 in LRG, which is associated with mucosal healing [19], might predict the potent anti-inflammatory effects of this targeted approach. Treatment with vedolizumab or upadacitinib was effective in the group with lower neutrophilic infiltration (Geboes < 3.2). This suggests that in the absence of intense epithelial neutrophil invasion, other pathways—such as lymphocyte trafficking (targeted by vedolizumab) or broader cytokine signaling (targeted by upadacitinib)—may be more dominant drivers of inflammation. This approach may allow for preservation of IL-23p19 inhibitors for patients with specific histopathologic characteristics, potentially optimizing outcomes across different patient subgroups. A central confounding factor in this study is the inability to distinguish the effects of our histology-based patient selection from those of the specific mirikizumab induction regimen administered. Our protocol directed patients with significant neutrophil infiltration (Geboes score ≥ 3.2) to receive mirikizumab, a strategy that resulted in notably high rates of clinical and endoscopic remission. However, the induction regimen for most of these patients—300 mg intravenously every four weeks for 24 weeks—was a prolonged schedule based on Japanese product labeling. This regimen differs substantially from the standard 12-week induction phase (300 mg at weeks 0, 4, and 8) approved in the United States and other regions.

This discrepancy precludes a definitive conclusion as to whether the observed efficacy was driven by the enrichment of patients with IL-23 pathway-dominant disease or by the prolonged, higher-frequency induction dosing. While both mechanisms are biologically plausible, their respective contributions are inherently confounded within our study design. Accordingly, these findings should be regarded as hypothesis-generating and interpreted with caution. Future randomized controlled trials are necessary to distinguish these effects, for instance, by comparing the standard 12-week induction regimen against the extended 24-week schedule within a histologically defined patient cohort.

Although a central pathology review was not performed in this study, interobserver bias was addressed using the Komagane evaluation method [11], a refined and clearly defined subclassification of the Geboes histopathology score. This study confirmed that the method demonstrated high consistency among pathologists (Kappa value = 0.83), indicating strong reliability, even among non-specialists. Using this standardized scoring system enabled the reproducible classification of neutrophilic infiltration and minimized interobserver variability in histopathological assessment. Thus, the robust reproducibility of the Komagane subclassification supports the reliability of the pathology-based group assignments and mitigates concerns about potential observer bias in this single-center study.

In this study, endoscopic remission was selected as a secondary endpoint, and as a result, treatment with selective IL-23p19 inhibitors resulted in a high rate of endoscopic remission (i.e., mucosal healing) in patients with UC. IL-23 plays a central role in the pathogenesis of UC, promoting Th17 cells. Th17 cells produce inflammatory cytokines (such as IL-17) and sustain chronic inflammation of the intestinal mucosa [20,21,22]. As shown in Table 3, conventional anti-TNFα antibodies, anti-Integrins, and Janus kinase inhibitors inhibit multiple pathways, but IL-23p19 inhibitors selectively inhibit the Th17 pathway, the “driver” of inflammation, resulting in deeper mucosal healing [23]. Previous clinical trial comparisons have also shown in phase III trials that IL-23p19 inhibitors achieve higher rates of clinical remission and endoscopic mucosal healing than other agents [24]. By selectively suppressing the core pathway of inflammation, IL-23p19 inhibitors offer a targeted therapeutic approach [21]. Therefore, it would be extremely useful to have a method to select a group of patients for whom IL-23p19 inhibitors are effective.

Mirikizumab is a monoclonal antibody that selectively inhibits the p19 subunit of IL-23. Unlike conventional agents in the same class (e.g., ustekinumab), which block both IL-12 and IL-23, mirikizumab does not suppress IL-12 [25]. Therefore, it is expected to more precisely suppress chronic inflammation while minimizing the impact on host defense mechanisms, such as anti-infective and antitumor immunity, which are mediated by IL-12 [26]. The safety profile of mirikizumab is considered comparable, or nearly equivalent, to that of other biologics (especially ustekinumab or vedolizumab) [27]. Notably, mirikizumab demonstrates a clearly favorable safety margin compared to Janus kinase inhibitors, with a lower risk of infections and serious adverse events [28]. When evaluated against anti-TNFα antibodies, the incidence of infections and severe adverse events for mirikizumab is similar or slightly lower [28]. Therefore, the combination of strong efficacy and favorable safety profiles makes IL-23p19 inhibitors excellent candidates for first-line treatment in moderate-to-severe inflammatory conditions [23].

**Table 3:** A comparison of the mucosal healing rates of advanced ulcerative colitis therapy agents.

Class/Agent	Targeted Pathway	Mucosal Healing Rate	Mechanistic Notes
Selective IL-23p19 inhibitors	IL-23 (p19 subunit)	High (30%+) [23,24]	Targets upstream of Th17 cells, highly specific.
Anti-TNF antibodies	TNF-α broader cytokines	Moderate (~20–25%) [23,28]	Blocks a key pro-inflammatory cytokine, less specific. Immunogenicity can limit long-term effectiveness.
Anti-Integrins (Vedolizumab)	Gut-selective cell trafficking	Moderate (~15–25%) [23,28]	Inhibits lymphocyte migration to gut.
JAK inhibitors (Tofacitinib)	JAK-STAT intracellular pathway	Moderate to Low [23,28]	Broad inhibition, can have higher infection risk.
IL-12/23 inhibitors (Ustekinumab)	IL-12/IL-23 (p40 subunit)	Moderate to High [23,28]	Less specific, partly overlaps with IL-23p19 agents but may affect broader immune functions.

JAK: Janus kinase.

The study did not use random treatment allocation. Patients with Geboes ≥ 3.2 received mirikizumab, while those with Geboes 3.1 received vedolizumab. The manuscript mentions the upadacitinib arm for severe cases, but this arm is not described in the registered ClinicalTrials.gov protocol, which lists only mirikizumab and vedolizumab. This discrepancy raises concerns about deviations from the original protocol. Additionally, since histology determined which drug each patient received, the effects of the drugs and patient selection are confounded and cannot be separated.

We acknowledge that fecal calprotectin is a valuable biomarker for assessing UC activity. However, it was not included in our current biomarker panel. Our study focused on histopathology-driven treatment stratification and the measurement of systemic inflammatory markers, such as CRP and LRG. We acknowledge that CRP values were reported in milligrams per deciliter (mg/dL) and that this differs from the more commonly used milligrams per liter (mg/L) units. The timing of CRP measurement was consistent; it was performed on the same day as the colonoscopy and histology assessments, as described in the methods section. Despite the absence of fecal calprotectin, we believe that combining histological assessment with systemic biomarkers provides useful complementary information.

This study has several important limitations. First, the single-center, prospective design and small cohort restrict the external validity and generalizability of our findings. The patient population from a single institution may not be representative of the broader UC population, and local referral patterns and standards of care could influence the results, introducing a potential for selection bias. Second, the study was not randomized, and it lacked a concurrent control group for the histology-stratified interventions. Third, no formal sample size calculation was performed prior to the study. Consequently, the analysis is exploratory, and the study may lack the power to detect subtle yet clinically meaningful differences between groups. This carries a substantial risk of a Type II error, whereby a true effect may be overlooked. Therefore, our results should be considered hypothesis-generating. Finally, the six-month follow-up period for the secondary endpoint of endoscopic remission was insufficient to evaluate the long-term durability of the response.

## 5. Conclusions

In conclusion, this preliminary study suggests that a treatment strategy guided by a Geboes Score Grade 3 histopathological subclassification for active UC is a promising approach to personalizing therapy. Using a threshold of ≥3.2 to select patients for IL-23p19 inhibitor therapy seemed effective in this small cohort. However, further validation in larger, prospective, randomized controlled trials is needed to clarify the clinical relevance of this biomarker-guided strategy. Future research could build on our findings by developing a composite scoring system that integrates this histopathological subclassification with non-invasive biomarkers, particularly fecal calprotectin and endoscopic scores. This multifaceted tool could provide a more comprehensive evaluation of inflammatory activity and improve the accuracy of personalized treatment strategies for patients with UC.

## Figures and Tables

**Table 1:** Clinical characteristics of the enrolled subjects (*n* = 42).

Geboes Score	Grade ≥ 3.2	Grade < 3.2	*p*
Number of patients	22	20	
Males/females, *n* (%)	10 (45)/12 (55)	9 (45)/11 (55)	0.99
Age, yrs; median (25th, 75th percentile)	43 (32, 53)	43 (31, 55)	0.98
Disease duration, years; median (25th, 75th percentile)	2.0 (1.0, 3.0)	2.0 (1.0, 7.8)	0.37
Disease extension, *n* (%):			0.99
Left-sided colitis	10 (45)	10 (50)	
Pancolitis	12 (55)	10 (50)	
CAI, median (25th, 75th percentile	7.5 (7, 9)	7 (6, 9)	0.56
NRS, median (25th, 75th percentile)	7 (6, 7)	7 (4, 8)	0.25
MES, median (25th, 75th percentile)	2 (2, 2.25)	2 (2, 2.5)	0.99
UCEIS, median (25th, 75th percentile)	5.5 (5, 6)	6 (5, 6)	0.17
Medication before induction therapy, *n* (%):			0.15
5-ASA + steroids	7 (32)	10 (50)	
Infliximab	7 (32)	4 (20)	
Ustekinumab	4 (18)	0	
Filgotinib	4 (18)	6 (30)	

CAI: clinical activity index; NRS: numeric rating scale (for bowel urgency); MES: Mayo endoscopic subscore; UCEIS: Ulcerative Colitis Endoscopic Index of Severity; 5-ASA: 5-aminosalicylic acid.

**Table 2:** Changes in blood biomarkers before and after 4 weeks of treatment.

Parameter (Mean ± SD)	Before	After	*p*
Geboes ≥ 3.2 Group (*n* = 22)			
Hemoglobin (g/dL)	13.1 ± 1.3	13.7 ± 1.4	0.36
Albumin (g/dL)	3.9 ± 0.52	4.1 ± 0.51	0.40
CRP (mg/dL)	1.5 ± 5.8	0.034 ± 0.03	0.12
LRG (µg/mL)	18.3 ± 3.6	11.0 ± 1.9	<0.001
Geboes < 3.2 Group (*n* = 20)			
Hemoglobin (g/dL)	13.6 ± 1.6	14.2 ± 1.4	0.44
Albumin (g/dL)	3.8 ± 0.32	4.1 ± 0.44	0.56
CRP (mg/dL)	2.1 ± 4.5	0.043 ± 0.04	0.33
LRG (µg/mL)	20.3 ± 4.1	15.0 ± 3.1	<0.001

CRP: C-reactive protein, LRG: leucine-rich α2glycoprotein.

## Data Availability

The data underlying this article will be made available upon reasonable request to the corresponding author.
